# Assessing pretreatment reactor scaling through empirical analysis

**DOI:** 10.1186/s13068-016-0620-0

**Published:** 2016-10-10

**Authors:** James J. Lischeske, Nathan C. Crawford, Erik Kuhn, Nicholas J. Nagle, Daniel J. Schell, Melvin P. Tucker, James D. McMillan, Edward J. Wolfrum

**Affiliations:** National Renewable Energy Laboratory, National Bioenergy Center, 15013 Denver West Parkway, Golden, CO USA

**Keywords:** Biomass, Biofuels, Pretreatment, Enzymatic digestibility

## Abstract

**Background:**

Pretreatment is a critical step in the biochemical conversion of lignocellulosic biomass to fuels and chemicals. Due to the complexity of the physicochemical transformations involved, predictively scaling up technology from bench- to pilot-scale is difficult. This study examines how pretreatment effectiveness under nominally similar reaction conditions is influenced by pretreatment reactor design and scale using four different pretreatment reaction systems ranging from a 3 g batch reactor to a 10 dry-ton/days continuous reactor. The reactor systems examined were an automated solvent extractor (ASE), steam explosion reactor (SER), ZipperClave^®^Reactor (ZCR), and large continuous horizontal screw reactor (LHR). To our knowledge, this is the first such study performed on pretreatment reactors across a range of reaction conditions and at different reactor scales.

**Results:**

The comparative pretreatment performance results obtained for each reactor system were used to develop response surface models for total xylose yield after pretreatment and total sugar yield after pretreatment followed by enzymatic hydrolysis. Near- and very-near-optimal regions were defined as the set of conditions that the model identified as producing yields within one and two standard deviations of the optimum yield. Optimal conditions identified in the smallest scale system (the ASE) were within the near-optimal region of the largest scale reactor system evaluated. The maximum total sugar yields for the ASE and LHR were $95\,\%$, while $89\,\%$ was the optimum observed in the ZipperClave.

**Conclusions:**

The optimum condition identified using the automated and less costly to operate ASE system was within the very-near-optimal space for the total xylose yield of both the ZCR and the LHR, and was within the near-optimal space for total sugar yield for the LHR. This indicates that the ASE is a good tool for cost effectively finding near-optimal conditions for operating pilot-scale systems. Additionally, using a severity factor approach to optimization was found to be inadequate compared to a multivariate optimization method. Finally, the ASE and the LHR were able to enable significantly higher total sugar yields after enzymatic hydrolysis relative to the ZCR, despite having similar optimal conditions and total xylose yields. This underscores the importance of mechanical disruption during pretreatment to improvement of enzymatic digestibility.

**Electronic supplementary material:**

The online version of this article (doi:10.1186/s13068-016-0620-0) contains supplementary material, which is available to authorized users.

## Background

During biochemical conversion of biomass to fuels and chemicals, thermal, chemical, and/or mechanical pretreatment is necessary to render biomass materials less recalcitrant and more susceptible to deconstruction via enzymatic hydrolysis [1, 2]. A common pretreatment process, and the one used in this study, employs dilute sulfuric acid to hydrolyze the hemicellulose fraction to monomeric xylose and xylo-oligomers. The dilute acid-pretreated solids, which contain a large fraction of cellulose, are then enzymatically hydrolyzed to soluble glucose using cellulase enzyme cocktails [3].

Scaling up pretreatment result from bench-scale reactors to pilot-, demonstration-, and commercial-scale reactors is of primary interest to researchers, commercial technology developers, investment bankers, and the US Department of Energy (DOE) [4, 5]. Bench-scale pretreatment experiments are often performed because they are easier, safer, and less resource intensive than using pilot- and demonstration-scale systems [5]. Differences in reactor design, solid concentrations, heating methods (indirect or direct), heating/cooling profiles, mixing characteristics, and the extent of pre-impregnation of water and/or catalyst/reactant in the biomass affect pretreatment performance, and ultimately the total sugar yield obtained after pretreatment and enzymatic hydrolysis [4, 5].

Literature often reports differences in pretreatment and enzymatic hydrolysis yields for the same biomass feedstock using the same nominal pretreatment reaction severity conditions (i.e., pH, temperature, and residence time). These differences in sugar yields under nearly identical reaction conditions can be directly attributed to differences in pretreatment reactor design [5–8]. Studies by Wang et al. [7] and Ciesielski et al. [8] compared pretreatment and enzymatic hydrolysis sugar yields, as well as structural and morphological analyses, of corn stover pretreated at the same dilute sulfuric acid pretreatment reaction conditions using three different pretreatment reactor systems (bench-scale batch steam explosion and ZipperClave reactors, and a mini-pilot-scale continuous 200 dry g/h horizontal reactor). Even though the targeted pretreatment conditions were the same and the chemical compositions of the pretreated solids materials were similar (within analytical limits), different cellulose hydrolysis yields were obtained upon subsequent enzymatic hydrolysis. These differences in enzymatic digestibility despite nominally identical reaction conditions underscore the importance of reactor configuration, indicating that either the stated conditions were not precisely achieved in each reactor, or there were physical transformations occurring in one configuration but not in another, or both. Understanding how pretreatment reactor configuration influences overall sugar yields is crucial for improved reactor design and for scaling up pretreatment technologies from the bench-scale to industrial-scale (and any intermediate scales in between).

Ropers et al. [9] examined steam explosion across a range of conditions for a bench-scale batch reactor and a pilot-scale continuous reactor. Comparisons between nominal conditions were made on the basis of severity factor and enzymatic hydrolysis yield. They found that these reactors had significantly different optimal conditions, underscoring the need for careful study of scale-up. However, their results may be confounded by inconsistent preprocessing of the material between the reactors: corn stover used in the pilot-scale reactor had been hammer-milled in order to facilitate material flow, whereas corn stover used in the batch reactor did not undergo any preprocessing.

While there have been other detailed studies comparing the experimental and techno-economic performance of leading pretreatment technologies, most notably those carried out by the former Biomass Refining Consortium for Applied Fundamentals and Innovation (CAFI) [10], to the authors knowledge, there are no studies beyond those already cited [7–9] that assess the impact of pretreatment reactor type and size (scale) for a given pretreatment chemistry. Accordingly, the goals of this work were to (1) analyze and explicitly compare the performance of four unique pretreatment reactors across a wide range of operating conditions using a common corn stover feedstock and dilute acid pretreatment chemistry and (2) determine how effectively the results from smaller-scale, higher-throughput systems can be used to guide and identify optimal pretreatment conditions in larger, pilot-scale, more process-relevant pretreatment reactor systems.

Four well-established pretreatment reactors were used, each operating at a different level of scale and process relevance and with a design of experiment appropriate to the scale and operating limitations of the equipment. These systems are as follows: an Accelerated Solvent Extractor 350 (ASE) [11], a steam explosion reactor (SER) [6], a ZipperClave reactor (ZCR) [6], and a large continuous horizontal screw reactor (LHR) [12]. The differences between the pretreatment reactors systems are summarized in Table 1 and more thoroughly described in the Material and Methods section. These reactors have throughputs ranging from grams-per-day (ASE) to hundreds of kilograms per day (LHR), which impact the labor and material costs of optimization study. Additionally, our smaller-scale systems require less time to run multiple conditions, and our smallest system, the ASE, is fully automated, which substantially reduces the cost of broad, multi-factor optimization studies.

**Table 1:** Summary of the reactor configurations and experimental conditions for the four pretreatment reactor systems used in this study

	ASE-350 (ASE)	ZipperClave^®^(ZCR)	Steam explosion (SER)	Large horizontal reactor (LHR)
Operating mode	Batch	Batch	Batch	Continuous
Sample amount (kg, dry basis)	0.003	0.07–0.10	0.25	10–25 kg/h
Operational capacity (kg/day)	0.03	0.7	0.8	600
Biomass impregnation	In situ	Ex situ	Ex situ	Ex situ
Heating	Oven	Steam injection	Steam injection	Steam injection
Minimum residence time (min)	4	4	1	10
Solids loading ($\%$)	10	25	25–30	30
Conditions per day$^\mathrm{a}$	9	9	8	4
Operator hours per condition$^\mathrm{b}$	0.5	2	2	6
Mechanical shearing	–	–	X	X
Rapid decompression	–	–	X	X

^a^Expected maximum based on full-time operation over several days

^b^Includes the time required to prepare, run, and shut down the equipment. Compositional analysis of output streams and yield calculations is not included

Furthermore, the modes of operation of the reactors are each quite different. Briefly, the ASE is an automated bench-scale system desirable for high-throughput pretreatment screening. However, it is oven-heated rather than heated by direct steam injection, resulting in a much longer heat-up time. At the end of reaction, liquor inside the ASE is drained at the pretreatment temperature and pressure, and then the pretreated biomass solids are flushed with water at $100 \,\,^\circ \mathrm {C}$. The ZCR and SER, on the other hand, are 1- and 4-L reactor vessels that are both rapidly heated by steam injection and cooled by releasing pressure inside the vessel. The ZCR, has a long pressure-release time (approximately 60 s), whereas the SER releases pressure suddenly (${<}1$ s), ejecting pretreated material through an extrusion die, which also results in an additional mechanical shearing of the pretreated solids. The LHR is a continuous screw-fed pretreatment vessel, which enables a range of potential residence times [13]. The LHR is heated by direct steam injection, and the material is ejected from this reactor using a flash valve airlock system, similar to the SER except without an extrusion die. We expect that these differences in reactor configurations and characteristics will impact the nominal time and temperature conditions of the reactors, confounding the definition of true reaction conditions and influencing the maximum achievable sugar release in each system.

## Results

An empirical design of experiment and optimization approach was used for each pretreatment reactor system. Reaction time varied from 5 to 25 min and temperature from 140 to $180\,\,^\circ \mathrm {C}$, depending on the limitations of the reactor system. Additionally, the experimental space included conditions previously shown to be optimal for similar biomass feedstocks. Acid loading for each reactor system was held constant at $1\,\%$ (on a mass per dry biomass basis) across all experiments and reactor systems.

The primary analytical data were used to determine various yields from the process. We are particularly interested in the total (that is, the sum of monomeric and oligomeric) xylose yield after pretreatment (hereafter referred to as total xylose yield), and the total glucose and xylose yield after pretreatment and enzymatic hydrolysis (hereafter referred to as total sugar yield). Reaction conditions and yields for the complete datasets are presented in Tables 2, 3, 4 and 5.

**Table 2:** Automated Solvent Extractor 350 system

Temperature ($\,^\circ \mathrm {C}$)	Residence time (min)	Log R$_0$ severity	Liquor pH	Total xylose yield (−)	Total sugar yield (−)
160	8	2.67	2	0.766	0.953
140	8	2.08	2	0.639	0.765
160	8	2.67	2	0.764	0.943
160	8	2.67	2	0.770	0.948
160	4	2.37	2	0.786	0.932
160	12	2.85	2	0.742	0.926
180	8	3.26	2	0.529	0.871
180	4	2.96	2	0.601	0.890
180	12	3.43	2	0.455	0.815
140	12	2.26	2	0.666	0.801
140	4	1.78	2	0.529	0.697
160	8	2.67	2	0.761	0.932
140	8	2.08	2	0.598	0.743
160	8	2.67	2	0.772	0.940
160	8	2.67	2	0.765	0.944
160	8	2.67	2	0.766	0.935
160	8	2.67	2	0.791	0.956
160	8	2.67	2	0.806	0.962
160	8	2.67	2	0.769	0.936

**Table 3:** Steam explosion reactor

Temperature ($\,^\circ \mathrm {C}$)	Residence time (min)	Log R$_0$ severity	Liquor pH	Total xylose yield (−)	Total sugar yield (−)
170	12.5	3.16	2.04	0.701	0.890
170	20	3.36	2.07	0.642	0.895
170	5	2.76	1.88	0.775	0.935
170	12.5	3.16	1.89	0.735	0.919
170	40	3.66	1.93	0.540	0.868
170	20	3.36	1.88	0.664	0.903
140	12.5	2.27	1.85	0.647	0.791
149	17.8	2.69	1.94	0.782	0.859
149	40	3.04	1.97	0.679	0.854
170	12.5	3.16	1.96	0.609	0.857
170	5	2.76	1.88	0.815	0.926
191.2	7.2	3.54	1.95	0.569	0.901
200	12.5	4.04	1.98	0.294	0.806
140	12.5	2.27	1.94	0.644	0.777
148.8	7.2	2.29	1.82	0.745	0.824
170	12.5	3.16	1.89	0.680	0.896
191.2	17.8	3.94	1.97	0.314	0.784
191.2	40	4.29	2.05	0.120	0.742
200	12.5	4.04	1.87	0.221	0.759
170	12.5	3.16	1.76	0.670	0.875

**Table 4:** ZipperClave reactor

Temperature ($\,^\circ \mathrm {C}$)	Residence time (min)	Log R$_0$ severity	Liquor pH	Total xylose yield (−)	Total sugar yield (−)
170	12.5	3.16	1.84	0.751	0.921
170	17.5	3.30	1.82	0.709	0.904
200	12.5	4.04	1.91	0.448	0.762
190	7.5	3.52	1.85	0.655	0.820
140	12.5	2.27	1.78	0.568	0.536
140	12.5	2.27	1.7	0.561	0.667
149	40	3.04	1.92	0.690	0.807
170	40	3.66	1.99	0.581	0.751
170	5	2.76	1.7	0.728	0.915
170	5	2.76	1.63	0.710	0.822
170	12.5	3.16	1.82	0.733	0.818
170	12.5	3.16	1.76	0.736	0.884
170	12.5	3.16	1.79	0.762	0.870
149	17.5	2.69	1.67	0.703	0.773
149	7.2	2.30	1.71	0.611	0.648

**Table 5:** Large horizontal reactor

Temperature ($\,^\circ \mathrm {C}$)	Residence time (min)	Log R$_0$ severity	Liquor pH	Total xylose yield (−)	Total sugar yield (−)
150	10	2.47	2.02	0.704	0.814
165	10	2.91	2.04	0.771	0.963
165	17.5	3.16	2.19	0.717	0.951
180	25	3.75	2.24	0.479	0.863
150	17.5	2.72	2.12	0.727	0.900
165	17.5	3.16	2.14	0.697	0.917
165	25	3.31	2.15	0.733	0.928
180	10	3.36	2.13	0.569	0.925
150	25	2.87	1.98	0.741	0.943
165	17.5	3.16	1.99	0.732	0.962
180	17.5	3.60	2.12	0.559	0.933

Total xylose yields ranged from approximately 0.45–0.80 (all yields are reported as fractional molar yield) for each reactor, except for the SER, which had one condition which produced a total xylose yield of 0.12. The highest yield was achieved in the SER, with a total xylose yield of 0.82 produced at $170\,\,^\circ \mathrm {C}$ and a residence time of $5\, \,\mathrm {min}$.

Total sugar yields ranged from approximately 0.70 to 0.92–0.96 for each reactor, with the notable exception that the LHR’s lowest total sugar yield was 0.81, produced at $150\,\,^\circ \mathrm {C}$ and a residence time of $10\,\,\mathrm {min}$, the least severe condition within that reactor’s experimental design. The LHR and ASE produced approximately equivalent maximum observed total sugar yields of 0.96 (using reaction conditions of $165\,\,^\circ \mathrm {C}$, $10\,\,\mathrm {min}$, and $160\,\,^\circ \mathrm {C}$, $8\,\,\mathrm {min}$ respectively), and the ZCR and SER generated maximum observed yields of 0.92 ($170\,\,^\circ \mathrm {C}$, $12.5\,\,\mathrm {min}$), and 0.93 ($170\,\,^\circ \mathrm {C}$, $5\,\,\mathrm {min}$), respectively.

To make a valid comparison across reactor systems at various operating conditions, it was important that reaction acid concentration was held constant. To that end, the same acid-impregnated feedstock was used in the ZCR, SER, and LHR, and in the ASE acid was added to the system to achieve approximately the same concentration. Also, pH was measured in each pretreated slurry (that is, at the reactor endpoint), and the pH of each was approximately $1.9\,\pm \,0.2$, which is near the limit of the precision of our pH measurement, verifying that acid concentration was held approximately constant between reactors.

A direct measure of the precision of the experimental data is the pooled standard deviation of replicate measurements. With one exception, the pooled standard deviations for each reactor system in each measure of merit are about $2\,\%$ (absolute) or less.

## Discussion

We are interested not only in the yields that can be enabled by each reactor, but also in the comparability of the optimal conditions identified for each reactor. Therefore, two modeling approaches are advanced. The first is based on the severity factor, a common tool in the literature for discussing pretreatment reaction conditions in a simplified way [14–16]. The second is a multivariate response surface modeling approach. Using optimal conditions in each reactor identified by these modeling approaches, we evaluate the comparability of the different pretreatment reactor systems.

### Severity factor modeling

For ease of comparing experimental results, pretreatment time and temperature are often combined into a single parameter known as the severity factor ($R_0$):

1 $$\begin{aligned} R_0 = t \times \mathrm {exp} \left( \frac{T-100}{14.75} \right) \end{aligned}$$

where *t* is the reaction time (in min), *T* is the reaction temperature (in $\,^\circ \mathrm {C}$), and 14.75 is an arbitrary constant based on the activation energy when assuming pseudo-first-order kinetics [17]. The severity factor has evolved beyond Eq. 1 to incorporate the addition of chemical catalyst (both at high and low pH) [14–16, 18]. However, this study used only a single pretreatment chemistry (dilute acid) and maintained the same acid concentration across all reactors. Therefore, we use the two-parameter severity model here, and present the data in terms of $\text{log}_{10}(R_0)$ for simplicity. This is used as a simple way to efficiently compare pretreatment effectiveness across multiple reactor systems and to suggest conditions suitable for scale-up.

**Fig. 1:**
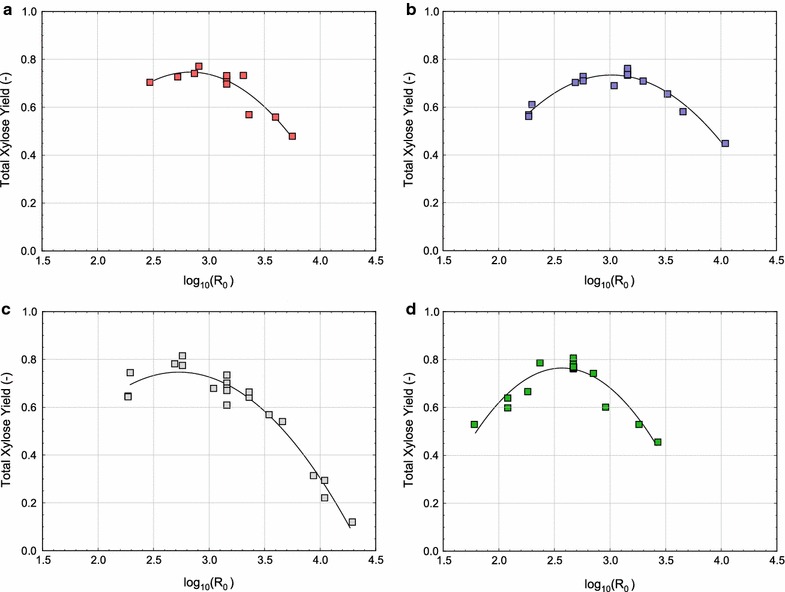
Total xylose yield after pretreatment as a function of severity factor $(\mathrm {log_{10}}(R_0))$ for the **a** LHR, **b** ZCR, **c** SER, and **d** ASE reactors.* Symbols* are experimental data and the *solid lines* are polynomial fits to guide the eye

**Fig. 2:**
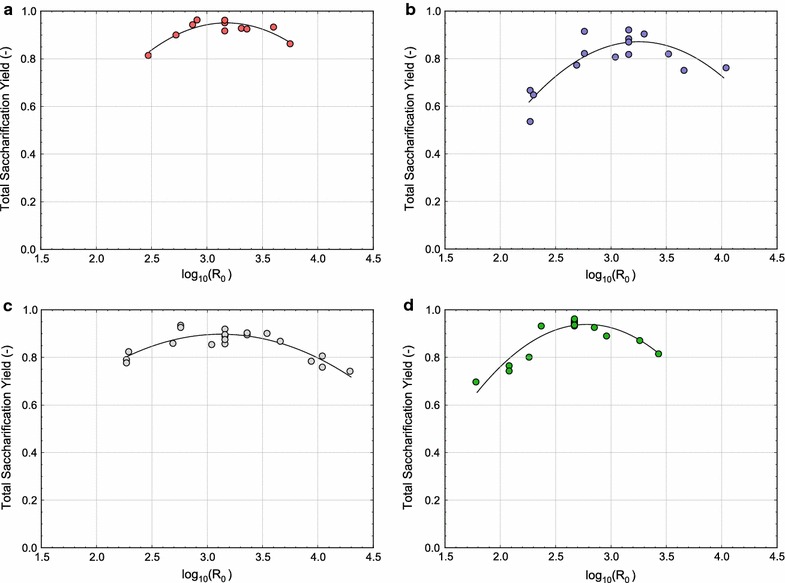
Total sugar yield after pretreatment and enzymatic hydrolysis as a function of severity factor $(\mathrm {log_{10}}(R_0))$ for the **a** LHR, **b** ZCR, **c** SER, and **d** ASE reactors.* Symbols* are experimental data and the *solid lines* are polynomial fits to guide the eye

Optimum pretreatment conditions for maximum total xylose yield (Fig. 1) and total sugar yield (Fig. 2) appear to have been reached for each of the reactor systems. The optimum $\text{log}_{10}(R_0)$ values ranged from 2.5 to 3.25 for each measure of merit, with the ASE requiring the lowest severity for optimum yield. The SER and LHR reactors required more severe pretreatment conditions to enable maximum sugar release, while the ZCR needed the most severe reaction conditions. However, for all reactor systems, the optimum severity to enable maximum total sugar yield in a given reactor was higher than the optimum severity for maximum total xylose yield during pretreatment. In effect, this suggests that one should pretreat beyond the optimum conditions based on total xylose release in order to maximize total sugar yield following enzymatic hydrolysis.

The total sugar yields from the LHR and SER reactors are less dependent on pretreatment severity (i.e., the curves in Fig. 2a, c are relatively flat). Since both of these reactors include additional mechanical deconstruction in the forms of mechanical shearing and rapid decompression, improved enzymatic digestibility may be less dependent on pretreatment chemistry. Therefore, the LHR and SER reactors have a larger range of severity conditions which can enable reasonably high sugar release than the ASE and ZCR systems. The ASE and ZCR systems, on the other hand, rely solely on the kinetics of the pretreatment reaction (i.e., time and temperature) for biomass deconstruction. As a result, the ASE and ZCR sugar yields are more strongly correlated with $\text{log}_{10}(R_0)$ (Fig. 2b and d), and are thus more likely to produce poor sugar yields when operating outside the optimum severity range.

Even though optimum pretreatment severities were found in each reactor system (Figs. 1, 2), presenting the data in this fashion can be slightly misleading. For instance, the LHR, ZCR, and SER display larger variances in their replicate points than the ASE (Fig. 2). Thus, the optimum pretreatment severity for the LHR, ZCR, and SER can be predicted much less precisely than for the ASE reactor (which adds some ambiguity to the identification of optimal conditions). In addition, while the SER data (Fig. 2c) suggest that an optimum pretreatment severity was reached during experimentation, multivariate modeling (discussed in the next section) rather suggests that optimal reaction conditions for the SER were not reached within the experimental design used in this work. Thus, while the severity factor concept may be useful for comparing overall performance between reactors at different operating conditions, it is not a robust technique to identify optimal operating conditions.

### Multivariate response surface modeling (RSM)

The experimental data were also used to generate empirical response surface model (RSM) contour maps describing the performance metrics as second-order polynomial functions of pretreatment reaction time and temperature [19]. Adjusted-R$^2$ is a measure of the quality of an empirical fit that takes the sample size and number of fit parameters into account. All adjusted-R$^2$ values for the ASE were above 0.95, but, for the other reactors, adjusted-R$^2$ ranged from 0.86 to 0.97 for monomeric and total xylose yields from pretreatment. The quality of fit was lower for total sugar yield in both the LHR (0.66) and the ZCR (0.80). These data, along with optimal conditions and yields, and the replicate standard deviation in each reactor, are shown in Table 6.

**Table 6:** Summary of pretreatment results

	ASE	ZCR	SER^a^	LHR
*Total xylose yield*				
Optimal yield	0.789	0.746	0.797	0.766
T °C	156	163	161	160
t (min)	10	18	6	7
Adjusted* R* ^2^	0.98	0.97	0.95	0.93
Replicate S.D.	0.016	0.012	0.039	0.018
*Total sugar yield*				
Optimal yield	0.955	0.889	0.922	0.951
T °C	162	173	173	168
t (min)	10	15	6	16
Adjusted* R* ^2^	0.99	0.80	0.94	0.66
Replicate S.D.	0.010	0.061	0.021	0.023

Optimal conditions are obtained using RSM, and the values of the optima, as well as their conditions, are presented below. The quality of fit, as measured by adjusted-*R* ^2^, and the standard deviation of the replicates are also presented

^a^Optima for the SER are the maximum values of the models within the experimental space rather than absolute maxima

We are interested in comparing yield maxima between reactors, and in assessing the degree to which the optimal conditions identified using a smaller bench-scale pretreatment reactor system (i.e., the ASE) can predict optimal conditions for a larger pilot-scale reactor system like the LHR. The RSMs can be used to identify optimal reaction conditions for each pretreatment reactor, defined as the combination of time and temperature that give maximum total xylose and/or total sugar yields. We should expect the optimal conditions in each reactor to be different; it would be a highly improbable result that any two optima are precisely the same. Thus, we need a method of determining whether an optimal condition in one reactor is close enough (within experimental error) to the optimum condition of another reactor, either to be used directly, or to be used as a starting point for further optimization in the new reactor.

**Fig. 3:**
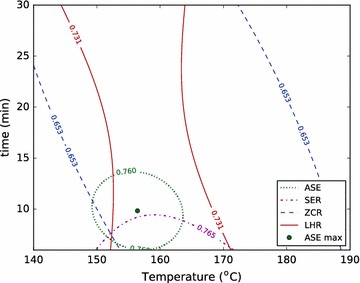
The RSMs are used, together with the standard deviations of replicate conditions in the ASE, SER, ZCR, and LHR to describe near-optimal spaces for each reactor for total xylose yield. The near-optimal space is defined as the conditions which produce a yield within two standard deviations of the optimal yield. The optimal condition found in the ASE is shown to illustrate the ability to directly use optimum conditions found in this high-throughput reactor as an approximation of the optimal conditions in larger reactor systems

**Fig. 4:**
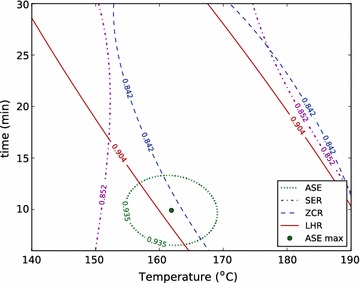
The RSMs are used, together with the standard deviations of replicate conditions in the ASE, SER, ZCR, and LHR to describe near-optimal spaces for each reactor for total xylose yield. The near-optimal space is defined as the conditions which produce a yield within two standard deviations of the optimal yield. The optimal condition found in the ASE is shown to illustrate the ability to directly use optimum conditions found in this high-throughput reactor as an approximation of the optimal conditions in larger reactor systems

To form a basis for these comparisons, we used the RSM data and the standard deviations of replicate conditions in each reactor to define near- and very-near-optimal spaces: we call any condition in the model which gives a yield within two standard deviations of the optimum a near-optimal condition and the set of these conditions the near-optimal space, because that result cannot be distinguished from the optimal result with a confidence of $95\,\%$. Similarly, we call the set of conditions that give results within one standard deviation of the optimum the very-near-optimal space. We then compared the optimal condition identified in the ASE to these near- and very-near-optimal spaces in other reactors (Figs. 3, 4).

The optimal values and conditions of the model equations for each reactor are given in Table 6. SER data are included on the basis of the maximum value of the model within the experimental space rather than the model equation’s absolute maximum, because the RSM returned optimal values well outside the range of validity. The optimal total xylose yield for the LHR, according to the RSM, is also outside the range of the experimental design (at 7 min, compared to a minimum residence time of 10 min). However, we choose to report this value as it is close to the range of validity, and it is possible to achieve this residence time by reconfiguring the reactor.

We note that total sugar yields for the LHR and the ASE were both above $95\,\%$ of theoretical at their respective optima, whereas the ZCR gave a lower value ($89\,\%$). Optimal total sugar yield for the SER was 0.92, but would likely be higher if the SER were allowed to operate at its optimal condition. This observation is consistent with our experience with these reactor systems [7, 8], which shows that the ZCR produced less-digestible pretreated material when compared to the SER and a horizontal screw reactor (of similar design to the LHR) when operated at the same targeted conditions. However, work presented here is the first to demonstrate this effect across a range of pretreatment conditions. The ZCR has a relatively long cool-down period, where pressure in the reactor is slowly released, in contrast to the rapid pressure release and cooling experienced in the SER and the LHR as a result of flashing the material as it exits the reactor. Flashing the material results in a sudden transition of liquid entrained within biomass particle cell walls to gas, which has been reported to significantly disrupt cell walls and lead to increased digestibility during enzymatic saccharification [7, 8, 20].

The ASE, like the ZCR, does not flash pretreated material at the end of reaction, but, like the LHR and SER, the ASE enables higher total sugar yields than the ZCR. The ASE does, however, separate the pretreated liquor from the solids at the pretreatment temperature and then rinse the solids with hot water. This may result in a lignin-extraction effect due to hot-washing [21, 22]; lignin is a well-known inhibitor of enzymatic digestion [23–25], and its removal has been previously shown to be responsible for increased enzymatic digestibility. Together, these data lend support to the idea that disruption of cell walls through steam explosion or lignin removal is an important part of preparing biomass for enzymatic hydrolysis. In this dataset, we see that steam explosion and lignin removal have approximately the same impact on improving digestibility [20].

Figures 3 and 4 show that the ASE gives a relatively narrow distribution of near-optimal conditions, while the other systems produce much larger near-optimal regions. This is largely due to the nature of the ASE system, which has the smallest standard deviation of replicates, and therefore the highest reproducibility (Table 6). In addition, we see that the optimal total xylose yield and total sugar yield are only weakly time-dependent for the ZCR and the LHR pretreatment systems. Again, we believe that the mechanical influences of direct steam heating and decompression, along with residence time distribution, which are not quantified, weaken the dependence on time of these optima.

Total sugar yield also has a broad range of near-optimal conditions for the ZCR, SER, and LHR. In the LHR, this is a reflection of the data. Only two of eleven conditions tested produced total sugar yield below $90\,\%$, and those were the lowest and highest severity conditions in the design ($150\,\,^\circ \mathrm {C}$ for 10 min, and $180\,^\circ \mathrm {C}$ for 25 min) (Additional file 1). All other conditions tested in the LHR produced over $90\,\%$ total sugar yield, and each of these conditions falls within the near-optimal space shown in Fig. 4. Additionally, the variance of all the data points within the near-optimal region was only slightly higher than the variance of the replicate measurements (3.1 versus $2.3 \,\%$ absolute). These results underscore the robust nature of dilute acid pretreatment at the pilot-scale, which can not only enable total sugar yields above $90\,\%$ over a relatively broad range of conditions, but they also show the significant variability associated with pilot-scale work. The combination of these factors—a robust process with high replicate variability—makes precise identification of optimal conditions difficult at the pilot-scale.

Importantly, though, the optimum condition of the ASE is within the very-near-optimal space for the total xylose yield of the LHR and ZCR reactors, and is within the near-optimal space for total sugar yield of the LHR and the very-near-optimal space of the SER. This indicates that the optimal conditions identified for both total xylose yield and total sugar yield in the ASE reactor were statistically similar to and predictive of those for other reactors, and particularly the LHR. This has important implications for the ASE’s potential for screening other materials where there is less experience and intuition to guide condition-selection at the pilot-scale: one may simply perform a broad screening study on the ASE at a fraction of the cost, use the resulting optimal condition directly in a pilot-scale reactor, and be confident that they are near optimal conditions.

Despite this statistical agreement, the ASE consistently slightly underestimated the optimal pretreatment residence time for the other reactor systems. This may be because the ZCR, SER, and LHR are heated by steam injection, which quickly heats the biomass (${<}1$ min), whereas the ASE oven-heats the biomass, which takes substantially longer time (6–8 min). Additionally, there is no thermocouple inside the ASE reactor chamber to indicate when it has reached reaction temperature, so the heating time is heuristically defined by the manufacturer based on the selected pretreatment temperature, and automatically assigned by the instrument’s methods. This may lead to underestimating the total reaction time because the long heat-up time is not included in the total reaction time for the ASE system. Thus, it may be reasonable to add a small amount of time (e.g., a correction of 2–4 min) to the optimal time determined using the ASE to account for the additional reaction severity achieved in the ASE system during heat up. Further mechanistic modeling is required to fully justify this approach.

**Fig. 5:**
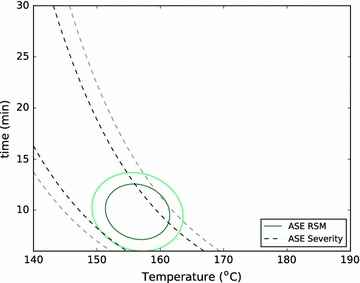
Near- and very-near-optimal zones are shown using a severity-modeling approach (*dashed*) and the multivariate RSM approach for total xylose yield in the ASE

**Fig. 6:**
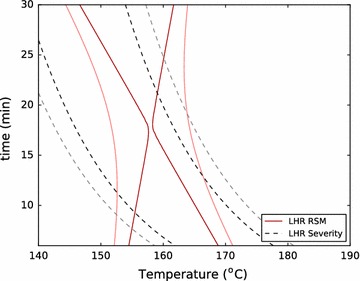
Near- and very-near-optimal zones are shown using a severity-modeling approach (*dashed*) and the multivariate RSM approach for total xylose yield in the LHR

The analysis shown in Figs. 3 and 4 can be repeated using the severity factor approximation in Eq. 1. Figures 5 and 6 show near- and very-near-optimal zones for total xylose yield in the ASE and the LHR, respectively. Using the severity factor approach predicts a much larger optimal zone for the ASE (Fig. 5) than obtained using RSM. That is, the severity factor approach incorrectly identifies many conditions as optimal that the RSM accurately describes as suboptimal. This clearly demonstrates the value of multivariate modeling over a combined-factor approach like severity.

Interestingly, these optimal zones predicted with the RSM and severity approaches match up better for the LHR (Fig. 6). That said, a comparison between an optimal severity factor range in the ASE and optimal conditions in the LHR shows that severity factor is less useful for identifying scale-up conditions than multivariate RSM.

Therefore, we suggest the following as guidance. Optimal operating conditions in a small-scale reactor such as the ASE, as determined using a multivariate optimization approach, may be used directly to identify near-optimal conditions for larger systems such as the LHR (i.e., the yield of the LHR at that condition is likely to be within two standard deviations of a true optimum yield). Due to the higher variability of larger-scale systems, severity factor may be used with caution as a first-approximation method to identify near-optimal conditions using experimental data produced in the same system, but it is an inherently inferior approach to multivariate modeling. Also, when designing production-level pretreatment reactor systems, physical transformations such as those produced by steam explosion and by lignin extraction should be considered alongside reaction kinetics.

## Conclusions

Response surface models describing total xylose yield after pretreatment and total sugar yield after pretreatment and enzymatic hydrolysis were developed for dilute acid pretreatment of corn stover carried out using four unique pretreatment reactor systems: an Accelerated Solvent Extractor 350 (ASE), a steam explosion reactor (SER), a ZipperClave reactor (ZCR), and a large continuous horizontal screw reactor (LHR). This cross-reactor study was performed using a single lot of corn stover and a single acid loading ($1\,\% \,\mathrm {w/w}$ sulfuric acid). Pretreatment monomeric and oligomeric xylose yields, and total sugar yields following pretreatment and enzymatic hydrolysis were determined. The pooled standard deviations for each system in each measure of merit were about $2\,\%$ (absolute) or less. These data were used to calculate optimal reaction conditions for each reactor system and identify experimental domains where each pretreatment reactor system achieves functionally equivalent results using both combined-severity factor and multivariate response surface modeling.

The calculated optimal conditions from the smallest reactor studied (the ASE) were then compared to these conditions in larger-scale reactors to investigate the possibility of predicting optimal conditions in the larger-scale reactors from the ASE results. The optimum condition identified in the ASE was within the very-near-optimal space for the total xylose yield of both the ZCR and the LHR, and was within the near-optimal space for total sugar yield for the LHR and the very-near-optimal space in the SER. Given the substantially lower cost of running screening studies on the ASE relative to pilot-scale reactors, this result indicates that the ASE is a good tool for finding approximately near-optimal pretreatment conditions in pilot-scale systems.

Additionally, though near-optimal experimental spaces are similar for the ZCR and the LHR, the LHR is able to enable higher total sugar yields, $95\,\%$ compared to $89\,\%$ in the ZCR, which reflects the importance of rapid decompression at the end of the pretreatment reaction. Although mechanisms for improved enzymatic digestibility are different for the LHR and the ASE (steam explosion versus lignin extraction), in this dataset they produced a similar impact on overall sugar yields achieved near optimal conditions. Finally, the RSM approach was compared to a combined-severity factor modeling approach. The combined-severity factor approach was less successful at identifying optimal reactor conditions, particularly in the ASE, where it incorrectly identified a broad range of suboptimal conditions as optimal.

## Methods

### Feedstock

We used a corn stover harvested in 2014 from Boone County, IA, provided by Idaho National Laboratory. The corn stover was milled using a knife mill (Jordan Reduction Systems, Birmingham, AL) to pass through a $20\,\,\mathrm {mm}$ round screen. For the ASE experiments and compositional analysis, a representative subsample of the $20\,\,\mathrm {mm}$ material was milled again in a smaller knife mill (Wiley, NY) to pass through a $2\,\,\mathrm {mm}$ screen.

### Acid impregnation

Acid impregnation was used in order to reduce reactor-to-reactor variability due to differing methods of acid addition. Dilute acid impregnation was performed in a 1900-L paddle mixer (American Process Systems, Gurnee, IL). Corn stover (120 dry kg) was added to the paddle mixer along with a dilute ($0.8\,\% \,\mathrm {w/w}$) sulfuric acid solution at a total solids loading of $8\,\% \,\mathrm {(w/w)}$ (solid:liquid ratio of 1:12). The slurry was mixed for 2 h at room temperature and then pumped to a continuous screw press (Vincent Corp. Model CP10, Tampa, FL) for dewatering to 45–50 % (w/w) total solids (TS). Three batches were prepared, mixed, and sampled. This material was used for pretreatments conducted in the ZipperClave, steam explosion, and large horizontal reactors, as described in the following sections. The ASE reactor experiments were performed with the as-received biomass without pre-impregnation. The composition of the feedstock and the acid-impregnated biomass is given in Table 7.

**Table 7:** Composition of raw and acid-impregnated corn stover feedstock used in this work (on a whole dry weight basis)

	Glucan (%)	Xylan (%)	Galactose (%)	Arabinan (%)	Lignin (%)	Ash (%)	Acetyl (%)	Total mass closure (%)
Corn stover	35.8	19.5	2.5	4.1	15.8	5.8	2.2	98.0
Acid-impregnated corn stover	39.5	21.1	1.6	2.4	18.7	2.6	1.6	96.0

### Pretreatment

The following is a description of the reactor systems used, and of the experimental design followed for each reactor. Experimental designs were chosen based on the physical limitations of each reactor and experience and intuition regarding ideal operating conditions for pretreatment of corn stover. The features of each reactor are summarized in Table 1, and the reaction conditions are given in detail in Additional file 1.

#### Automated Solvent Extractor 350

The Automated Solvent Extractor 350 (ASE) has been used as an automated high-throughput, laboratory-scale, batch-mode pretreatment reactor [11]. A fixed volume of dilute acid ($1\,\% \,\mathrm {(w/v)}$ sulfuric acid, $30\,\,\mathrm {mL}$) is contacted with a fixed mass of biomass (3 g) in a $66\,\,\mathrm {mL}$ zirconium reactor vessel. The cell is oven-heated to a fixed temperature and then held at that temperature for the reaction time. At the end of this time, the reactor vessel is cooled, the liquor in the vessel is expelled and collected, and the biomass is rinsed with approximately 100 mL of de-ionized water at $100\,\,^\circ \mathrm {C}$. The resulting liquor streams are analyzed for soluble carbohydrates, acids, and lignin, and the rinsed solids are also retained for an enzymatic hydrolysis assay. Because the mass of the sample is quite small, no compositional analysis of the pretreated solid is performed prior to enzymatic hydrolysis.

A full-factorial design at three temperatures (140, 160, $180\,\,^\circ \mathrm {C}$) and three static hold times (4, 8, $12\,\,\mathrm {min}$) was used for the ASE 350 experiments. A total of 20 experiments were performed in two batches, with the center point ($160\,\,^\circ \mathrm {C}$, 8 min) replicated five times for each batch. Two other experimental conditions were repeated across the two batches.

#### Steam explosion reactor

The Steam Explosion Reactor (SER) is a jacketed 4-L pressure vessel designed for rapid investigation of multiple pretreatment conditions at a medium scale in batch mode [6]. After the reactor is pre-heated, pre-impregnated biomass ($500\,\,\mathrm {g}$) is loaded into the reactor. The system is quickly brought to the reaction temperature by steam injection (approximately 5–$10\,\,\mathrm {s}$, as measured by thermocouples near the top and bottom of the reactor). The temperature is maintained using a pressure-control valve to regulate the steam-supply pressure, and by electrical heating blankets on all exposed surfaces for the duration of the reaction time. At the end of this time, the steam is shut off, and the bottom valve is quickly opened, explosively discharging the pretreated solids into a 55-$\,\mathrm {gal}$ nylon or polypropylene HotFill^®^bag inside a 200-$\,\mathrm {L}$ flash tank. The pretreated slurry is then weighed and analyzed for insoluble solids fraction, the solids are analyzed for structural carbohydrates and lignin, and the liquors are analyzed for soluble carbohydrates, acids, and lignin.

A two-factor, two-level central composite response surface experimental design with five replicates at the center point condition (17 total pretreatment runs) was conducted in the steam explosion reactor. The two factors varied were again reaction time and temperature, with the center point condition being $170\,\,^\circ \mathrm {C}$ and 12.5 min.

#### ZipperClave^®^Reactor

The ZipperClave^®^Reactor (ZCR) (Autoclave Engineers, Erie, PA) is a jacketed 1-L pressure vessel designed for rapid investigation of multiple pretreatment conditions at a medium scale in batch mode [6]. It differs from the SER primarily by the means of cooling the reactor. Pre-impregnated biomass ($100\,\,\mathrm {g}$) is loaded into a canister and inserted into the ZipperClave body, and then both top and bottom segments are closed and locked. The system is brought to the reaction temperature by steam injection (approximately 20–$40\,\,\mathrm {s}$, as measured by thermocouples near the bottom and middle of the reactor vessel). The temperature is maintained using a pressure-control valve to regulate the steam-supply pressure for the duration of the reaction time. At the end of this time, the steam pressure is slowly released through a condenser over a period of 15– $30\,\,\mathrm {s}$ to eliminate boil-over, while still allowing for steam escape to reduce slurry dilution by condensate. At the conclusion of the experiment, the slurry, solids, and liquors were analyzed as in the SER reactor.

A central composite design was used for reaction time and temperature for the ZCR experiments, with four replicates at the center point condition. Reaction temperature ranged from 140 to $191\,\,^\circ \mathrm {C}$, and reaction time from 5 to $40\,\,\mathrm {min}$, with the center point condition being $170\,\,^\circ \mathrm {C}$ and 12.5 min.

#### Large horizontal reactor

The large horizontal reactor (LHR) (Metso Inc., Norcross, GA) is a 1 dry-ton/days horizontal tube reactor designed for pilot-scale continuous pretreatment [12]. Pre-impregnated biomass is fed into the reactor by a plug-screw feeder, and heated by steam injection at the entrance of the reactor. Residence time is controlled by the rotation rate of screws inside the reactor, which push the biomass along the reactor tube; the reported residence time in this work is a simple function of the length of the tube and the screw speed. For simplicity, the impact of residence time distribution in the LHR is not directly examined in this study, and residence time comparisons are made only on the basis of nominal residence time (i.e., the residence time set by the control system for each reactor). At the end of the length of the reactor, the material is discharged through a flash valve, which achieves rapid decompression and cooling of the biomass, as in the SER. After steady-state conditions were achieved and maintained for $20\,\,\mathrm {min}$, pretreated slurry was collected in a 55-$\,\mathrm {gal}$ drum for $30\,\,\mathrm {min}$, which was then sealed to prevent loss of hot vapor. The resulting slurry, solids, and liquors were then analyzed as in the SER and ZCR. Samples of flash vapor and a reactor bleed stream were also collected and measured for furfural and acetic acid concentrations.

A two-factor, three-level factorial design with three replicates at the center point condition (11 total pretreatment runs) was conducted in the LHR. The two factors varied in the study were again reaction time and temperature. The three temperature levels tested were 150, 165, and $180\,\,^\circ \mathrm {C}$, and the residence times tested were 10, 17.5, and 25 min.

### Analytical methods

Pretreated liquor was analyzed for soluble monomeric and total sugars, and soluble acids (i.e., HMF and furfural), and pretreated solids were analyzed for cellulose, xylose, and lignin contents except where noted [26, 27].

For the enzymatic hydrolysis assay, pretreated materials were washed with de-ionized water, then the hydrolysis was carried out at a $2\,\% \,\mathrm {(w/w)}$ solid loading using CTec2^®^(Novozymes, NC) at an enzyme loading of 20 mg cellulase protein/g dry pretreated washed solids. Enzymatic hydrolysis proceeded for six days at $50\,^\circ \mathrm {C}$, and at the end, the samples were filtered and the liquids were analyzed for soluble sugars.

Details related to the calculation of yields are presented in additional files.

## Additional file

10.1186/s13068-016-0620-0 Assessing pretreatment reactor scaling through empirical analysis.

## Abbreviations

ASE: Automated Solvent Extractor
SER: steam explosion reactor
ZCR: ZipperClave Reactor
LHR: large continuous horizontal screw reactor
RSM: response surface model
