# Supplementary material for: Assessing pretreatment reactor scaling through empirical analysis
Source: Biotechnol Biofuels. 2016 Oct 10;9:213. doi: 10.1186/s13068-016-0620-0 (PMC5057393; doi:10.1186/s13068-016-0620-0)
Supplement: Supplementary file 1 — 10.1186/s13068-016-0620-0 Assessing pretreatment reactor scaling through empirical analysis. [file 13068_2016_620_MOESM1_ESM.pdf]

Assessing pretreatment reactor scaling through empirical analysis – Additional Files

Reactor and Yield Calculation Details

The reactors are described in greater detail in the papers referenced for each. For the reader's convenience, the details related to yield calculations are described here.

Automated Solvent Extractor

This reactor is presented in detail in Wolfrum, et al 2013. Known mass of corn stover of known composition is placed in the reactor vessel, known volumes of acid and water are added to the system, and the system is heated to the reaction temperature and held for the specified times. At the end of the reaction, the liquid is purged, and then the remaining solids are washed. The resulting liquors are analyzed for sugar and acids (HMF and furfural) concentrations, as described in the methods section.

Using this concentration data, we calculate the mass sugars released, and compare that mass, with an anhydrous correction, to the mass of the xylose and glucose components of the corn stover to calculate glucose, xylose, and total sugar yields from pretreatment. This is summarized by the equation

$$\zeta_i = \frac{c_i V \eta_i}{x_i m} \tag{1}$$

where i is the sugar (xylose or glucose), ζ is fractional molar yield, c_i is the concentration of the sugar in the liquor in g/L, V is the volume of the liquor generated, η_i is the anhydrous correction factor (that is, the ratio of the molecular weight of the free sugar to that of the monomer), x_i is the mass-weighted fraction of the corn stover consisting of the sugar polymer, and m is the mass of the corn stover sample.

ZipperClave Reactor

A known quantity of acid-impregnated corn-stover, of known composition and solids fraction, is introduced to the reaction vessel. The system is heated by steam injection up to the reaction temperature, as measured by thermocouples at the center of the reaction vessel. At the conclusion of the reaction, the system is slowly cooled, and the pretreated material is removed. The solids fraction and liquor composition is determined using analytical methods previously described. The yield is calculated according to the equation:

$$\zeta_i = \frac{c_i m_t (1 - f_{is}) \eta_i / \rho_l}{x_i m_s} \tag{2}$$

where ζ_i, c_i, η_i and x_i are defined as in the ASE section, m_t is the total mass after reaction, f_{is} is the mass fraction of the insoluble solids, ρ_l is the density of the liquid, and m_s is the mass of the solids initially placed in the reactor.

Steam Explosion Reactor

A known quantity of acid-impregnated corn-stover is added to the reactor vessel, and steam is allowed to enter the reactor until the system reaches the reaction

temperature, as measured by the pressure of the system and verified by two thermocouples. After system has been held at the reaction temperature for the specified time, a flash valve is opened, and the slurry is forced through an extrusion die into a collection vessel. After the pretreated slurry is cooled to room temperature, the percent insoluble solids of the slurry and the density of the liquid is determined. Using compositional data from the liquors, the mass of soluble sugars can be found. This mass is divided by the mass of the polymeric sugars, with the anhydrous correction factor, to give molar yield. The following is a compact calculation:

$$\zeta_i = \frac{c_i m_t (1 - f_{is}) \eta_i / \rho_l}{x_i m_s} \tag{3}$$

where ζ_i, c_i, η_i and x_i are defined as in the ASE section, m_t is the total mass after reaction, f_{is} is the mass fraction of the insoluble solids, ρ_l is the density of the liquid, and m_s is the mass of the solids initially placed in the reactor. Note that this is essentially the same expression as for the ZipperClave reactor.

Large Horizontal Reactor

The reactor is presented in detail in Shekiro, et al, 2014. Yield is calculated on the basis of mass flow rates in a similar manner as above. The expression for the yield of sugar i is:

$$\zeta_i = \frac{c_i \dot{m}_{l,out} \eta_i / \rho_l}{x_i \dot{m}_{s,in}} \tag{4}$$

where ζ_i, c_i, η_i and x_i are defined as in the previous sections. The mass-flow of pre-impregnated corn-stover of known solids fraction ($f_{is,in}$) and composition is measured by a weigh-belt. The all solids in the system are accounted for by the weigh belt, and therefore our expression for the mass flow rate of solids into the system is:

$$\dot{m}_{s,in} = \dot{m}_{t,in} f_{is,in} \tag{5}$$

where $\dot{m}_{t,in}$ is the mass flow rate as measured by the weigh-belt system, and $f_{is,in}$ is the insoluble solids mass fraction of the acid-impregnated biomass.

In order to calculate the liquid mass flow rate, we add the mass flow rate of saturated steam into the system, which is measured by a flow meter, to the mass flow rate of the liquid in the acid impregnated biomass, and subtract the amount of water that exits through the flash vent. This last value is calculated by using the heat capacity of steam, the difference between the reactor and flash tank temperatures, and the heat of vaporization.

$$\dot{m}_{l,out} = \dot{m}_{t,in}(1 - f_{is,in}) + \dot{m}_{steam} - \dot{m}_{flash} \tag{6}$$

Enzymatic Hydrolysis and Total Yield

For the ZCR, SER, and LHR, pretreated solids were washed and dewatered, and the resulting solids were analyzed for solids content and composition. These solids were

diluted to form 2% solids (w/w) solutions, heated to 50°C, and cellulase (Novozymes C-TEC2 lot # $VCPI0007$, 20 mg/g-cellulose loading) was added to the system. A sodium-citrate buffer was also added to maintain the system at a pH of 4.8. The system was held at this condition for seven days, and the liquors were sampled and tested for composition.

Sugar yields from enzymatic hydrolysis were calculated on the basis of the mass of sugar in solution at the end of reaction compared to the mass of sugars entering the system with the solids. To calculate the mass of sugars, we assume that, at the end of hydrolysis, the solids are negligible in calculating total volume, and we assume that the density of the liquor, which is a dilute sugar solution, is 1.0 g/mL. Thus, the mass of sugar i produced is given by:

$$m_{i,out} = c_i * m_t / \rho_l \tag{7}$$

where c_i is the concentration of sugar i in the liquor, m_t is the total mass of the system, and ρ_l is the density of the liquor, which we assume to be 1.0 g/mL. The mass of sugar i initially in the solids is given by:

$$m_{i,in} = \gamma_i m_t f_{is,0} \tag{8}$$

where γ_i is the percent of the pretreated solids consisting of the polymeric sugar i, and $f_{is,0}$ is the concentration of solids in the system initially, which is simply 2.0%. Finally, the molar component conversion due to enzymatic hydrolysis is given by:

$$\xi_i = \frac{c_i \eta_i / \rho_l}{\gamma_i f_{is,0}} \tag{9}$$

where constants are defined as above, and η_i is the ratio of molecular weights, as in the pretreatment section.

For the ASE, not enough material is produced per pretreatment condition to successfully test the solids composition. Therefore, the solids composition is determined implicitly for this calculation:

$$x_{i,ASE} = \frac{x_i(1-\zeta_i)}{1 - x_{glu}\zeta_{glu} - x_{xyl}\zeta_{xyl}} \tag{10}$$

Total yield across pretreatment and enzymatic hydrolysis for a component is given by the equation:

$$\chi_i = \zeta_i + (1-\zeta_i)\xi_i \tag{11}$$

and the combined yield of xylose and glucose across pretreatment and enzymatic hydrolysis is given by:

$$\chi_t = x_{xyl,0}(\zeta_{xyl} + (1-\zeta_{xyl})\xi_{xyl}) + x_{glu,0}(\zeta_{glu} + (1-\zeta_{glu})\xi_{glu}) \tag{12}$$
